# Insertion compounds and composites made by ball milling for advanced sodium-ion batteries

**DOI:** 10.1038/ncomms10308

**Published:** 2016-01-18

**Authors:** Biao Zhang, Romain Dugas, Gwenaelle Rousse, Patrick Rozier, Artem M. Abakumov, Jean-Marie Tarascon

**Affiliations:** 1 Chimie du Solide-Energie, FRE 3677, Collège de France, 11 Place Marcelin Berthelot, Paris, Cedex 05 75231, France; 2 Réseau sur le Stockage Electrochimique de l'Energie (RS2E), FR CNRS 3459, Amiens 80039, France; 3 ALISTORE-European Research Institute, Amiens 80039, France; 4 Sorbonne Universités—UPMC Univ Paris 06, 4 Place Jussieu, Paris F-75005, France; 5 University of Toulouse III Paul Sabatier, CIRIMAT CNRS UMR 5085, 118 route de Narbonne, Toulouse, Cedex 09 31062, France; 6 EMAT, University of Antwerp, Groenenborgerlaan 171, Antwerp B-2020, Belgium; 7 Skolkovo Institute of Science and Technology, Moscow 143025, Russia

## Abstract

Sodium-ion batteries have been considered as potential candidates for stationary energy storage because of the low cost and wide availability of Na sources. However, their future commercialization depends critically on control over the solid electrolyte interface formation, as well as the degree of sodiation at the positive electrode. Here we report an easily scalable ball milling approach, which relies on the use of metallic sodium, to prepare a variety of sodium-based alloys, insertion layered oxides and polyanionic compounds having sodium in excess such as the Na_4_V_2_(PO_4_)_2_F_3_ phase. The practical benefits of preparing sodium-enriched positive electrodes as reservoirs to compensate for sodium loss during solid electrolyte interphase formation are demonstrated by assembling full C/P′2-Na_1_[Fe_0.5_Mn_0.5_]O_2_ and C/‘Na_3+*x*_V_2_(PO_4_)_2_F_3_' sodium-ion cells that show substantial increases (>10%) in energy storage density. Our findings may offer electrode design principles for accelerating the development of the sodium-ion technology.

## Abstract (web summary)

New sodium-ion battery technology requires better control over solid electrolyte interface formation. Here, the authors report a series of ball-milled sodium alloys and enriched insertion electrodes, which act as sodium reservoirs compensating for sodium loss during solid electrolyte interface formation.

## Graphical abstract

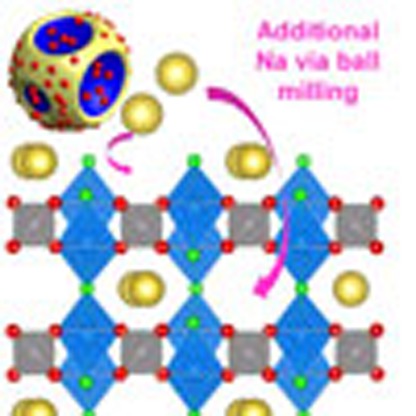

Considering elemental abundance, the most appealing alternative to Li-based battery technology is undoubtedly sodium. This is reflected in the revival of Na-ion battery (NIB) research, a field in which intense efforts are currently devoted to the search for high-performance electrode materials. Progress in sodium intercalation chemistry is primarily inherited from work on Li-ion materials, with the negative and positive electrodes for sodium systems based on similar structural types^1^,^2^,^3^. For negative electrodes comprising sodium metal, Na_*x*_Sb is the most attractive^4^. Antimony, however, is not the most desirable component considering its scarcity, moderate toxicity and large mass. There is thus great interest in carbon negative electrodes, whose reversible capacity can reach 300 mAh g^−1^ after accounting for a ∼25% irreversibility penalty associated with formation of the solid electrolyte interface (SEI) in the first cycle^5^. Among the positive electrode candidates, polyanionic compounds such as Na_3_V_2_(PO_4_)_2_F_3_ (NVPF) (refs 6, 7) and Na_2_Fe_2_(SO_4_)_3_ (ref. 8), and layered compounds like the O3-NaNi_0.5_Mn_0.5_O_2_ (ref. 9) and P2-Na_0.67_[Fe_0.5_Mn_0.5_]O_2_ (ref. 10) phases are presently the most studied. Nevertheless, the performances of NIB prototypes based on the aforementioned materials suffer from the large irreversibility of initial Na-uptake–removal processes at the carbon negative electrode.

The sodium loss in the formation of the SEI is similar to what is observed in the case of Li-ion batteries (LIBs), which has been widely studied. Because the only source of Li in the Li-ion cell is the Li-bearing electrode, compensating for lithium loss in the formation of the SEI at the negative electrode has been of great importance for achieving high energy densities in LIBs, and it is becoming more urgent with the emerging Si anodes, which show larger initial irreversibility than carbon electrodes. Through the years various routes have been explored. By adding to the positive electrode either sacrificial lithium species or additional intercalation compounds, the full capacity of a cathode material can be realized with modest weight penalties, For example, Li_2_C_2_O_4_ is used as a sacrificial source that decomposes during oxidation to provide extra lithium^11^. Alternately, in compounds such as Li_1+*x*_Mn_2_O_4_, which contains two redox voltages, the low-voltage ∼3 V plateau associated with *x*Li^+^ can be used as a Li reservoir^12^. Another approach, mainly applied to Si, is *in situ* or *ex situ* pre-lithiation. This can be accomplished either by Li ball milling^13^, or by placing Si in contact with Li so that when electrolyte is added formation of the SEI occurs conjointly with the uptake of Li by the negative Si electrode (Li_*x*_Si) (ref. 14).

Considering the lower coulombic efficiency of hard carbon in NIBs, that is, <80% (ref. 15), a strategy to compensate for Na loss to the SEI is sorely needed. It is also equally important to tune the degree of sodiation in cathodes to be used in NIBs, especially in the case of ‘sodium deficient' P2-type layered oxide electrodes of formula Na_2/3_MO_2_ (M=Mn, Fe, Co...)^10^,^16^, which only contain ∼0.67 Na^+^ per formula unit. These materials can achieve performances as high as 200 mAh g^−1^ but only in half cells against sodium metal which, in excess, enables NaMO_2_ compositions to be reached upon cycling. In contrast, the capacity drops by 30% in Na-ion cells because of the lack of a Na reservoir to compensate for the missing 0.33 Na^+^ in the pristine P2 phase. Solving these two issues constitutes the driving force of the present study.

Surprisingly few studies have been conducted before now to address the development of Na reservoir sources and pre-sodiation reagents for NIBs while minimizing energy penalty. Recently, NaN_3_ was proposed as a sacrificial Na source, but the generation of N_2_ gas during its non-reversible electrochemically driven decomposition upon oxidation is detrimental to the battery. Moreover, the low Na content in NaN_3_ (35% Na by weight) imposes a penalty in energy density^17^. Similar to LIBs, Na metal would obviously be the best pre-sodiation reagent for NIBs. However, it is one of the most difficult metals to handle because of its ductility and tendency to stick to metallic surfaces. Following our previous work on preparing Li-alloy through ball milling^18^, we report herein the synthesis of Na-based alloys and other insertion compounds via the ball milling of Na-metal with the appropriate chemical elements. We find this approach equally suitable for tuning the amount of Na in insertion electrodes. In particular, we find that Na_3_P-the Na-based compound showing the closest capacity (804 mAh g^−1^) to Na metal (1,165 mAh g^−1^) and hence offering the minimum weight penalty-can be used as a sacrificial Na source, increasing for instance the reversible capacity of C/P′2-Na[Fe_0.5_Mn_0.5_]O_2_ cells by 20%. We begin by describing the ball milling synthesis of Na-alloys, then present the use of the Na-ball milling approach for pre-sodiation of insertion positive electrodes (P2-Na_0.67_[Fe_0.5_Mn_0.5_]O_2_, NVPF), and end with the implementation of both approaches towards the optimization of full Na-ion cells.

## Results

### Ball milling-driven formation of Na-based alloys

**Figure 1:**
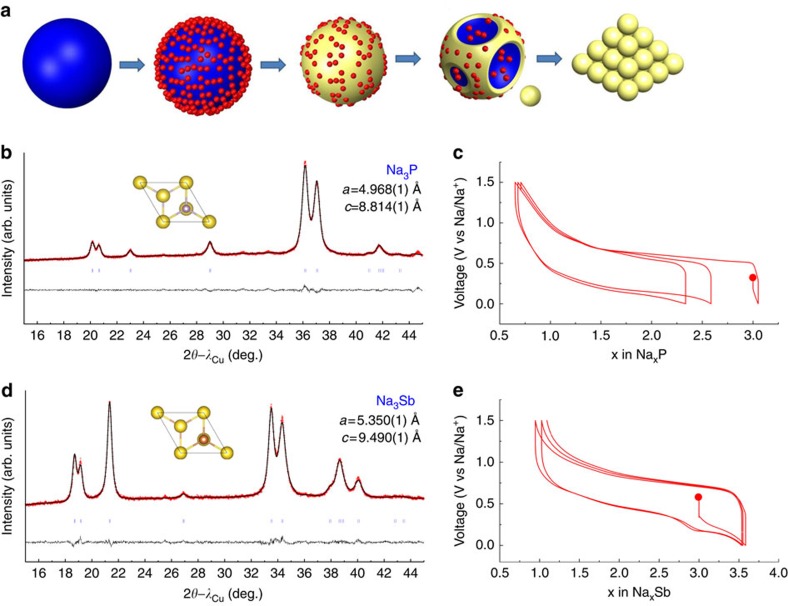
Synthesis of Na-based alloys. (**a**) Schematic diagram showing the ball milling process exemplified for the formation of Na_*x*_M alloys (yellow) by reacting Na (blue) with metal M (M=P or Sb) (red). In **b**,**c** and **d**,**e**, the Rietveld refined X-ray powder patterns of Na_3_P and Na_3_Sb powders are respectively shown together with their corresponding electrochemical voltage-profile in Na-half cells.

Na_3_P powders were prepared by adding stoichiometric amounts of Na-lumps and red phosphorous powder into a hardened steel ball milling jar, loaded in an Ar-filled glove box, with a ball/powder weight ratio of 35. Strikingly, room temperature continuous ball milling for 2 h, using a SPEX 8000 milling apparatus, was found to be sufficient to produce well crystalline and single-phased Na_3_P powders as deduced by X-ray diffraction (XRD) (Fig. 1a,b), while shear grinding for 27 h in planetary ball mill was shown to give a mixture of Na_3_P and amorphous phase^19^. These powders were mixed with carbon SP (30% by weight) and further ball milled for 20 min to obtain homogeneous composite electrodes. When cycled versus Na^+^/Na^0^, these composites showed reversible capacity (Fig. 1c) of ∼600 mAh g^−1^ based on the weight of Na_3_P.

To highlight the simplicity of this ball milling synthesis we recall the two approaches previously adopted to prepare Na_3_P powders: (i) electrochemical sodiation of P by making a battery using P and Na metal as working and counter electrode^20^, respectively; and (ii) chemical alloying of Na and P, either through a solvothermal reaction at 150 °C (ref. 21), or high temperature annealing (480 °C) in evacuated silica ampoules^22^. Encouraged by these results, we successfully extended the approach to the synthesis of Na_*x*_M alloys, where M is selected from the group consisting of Sn, Sb and Pb. For reasons of conciseness, the preparation of single-phase Na_3_Sb powders (Fig. 1d) whose voltage-composition curves fully mirror previous literature data (Fig. 1e)^23^ is solely reported herein.

Although speculative, we believe that the key to success for such unexpected reactions depends on the large free energy (Δ*G*) of the alloy formation reaction. As depicted in the schematic representations of Fig. 1a, a monolayer of alloy is rapidly created by contact of metal particles with Na lumps, hence separating the Na from the metal container and balls. Ball milling will continuously break the alloy shell to expose fresh surfaces, which rapidly react in a progressive alloying of all the Na, most likely facilitated by local heating. The repeated alloying/fracturing sequences are accompanied by a continuous peeling and breaking of the alloy shell into loose powders.

### Ball milling-driven pre-sodiation of insertion electrodes

**Figure 2:**
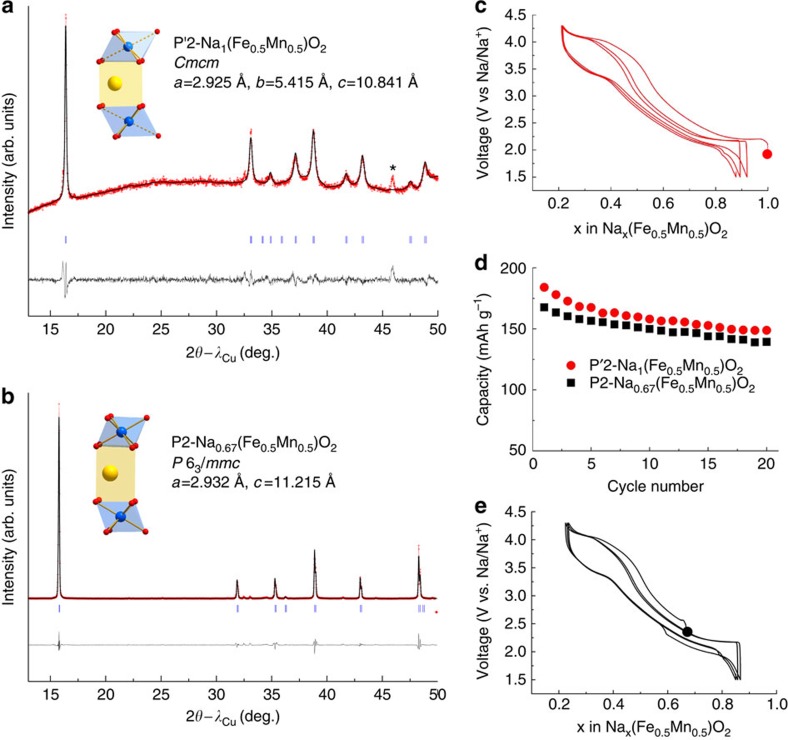
Synthesis of the P′2 phase by Na ball milling. XRD powder pattern profile matching and schematic representation of the structure of (**a**) P′2-Na_1_[Fe_0.5_Mn_0.5_]O_2_ obtained using ball milling with Na and (**b**) pristine P2-Na_0.67_[Fe_0.5_Mn_0.5_]O_2_. The refined cell parameters are in agreement with reported ones for both P2 and P′2 phases. The change of symmetry from P2 to P′2 accounts for the distortion of the MnO_6_ octahedral due to the Jahn–Teller effect of reduced Mn^3+^; voltage profiles of (**c**) P′2-Na_1_[Fe_0.5_Mn_0.5_]O_2_, (**e**) P2-Na_0.67_[Fe_0.5_Mn_0.5_]O_2_ and their corresponding cycling performances in Na-half cells (**d**). The peak marked with an asterisk (*) in **a** is attributed to Be window used to measure such a moisture sensitive sample.

The successful synthesis of Na-based alloys by room temperature Na ball milling encouraged us to exploit the reducing power of both Na and Na_3_P to simulate electrochemical reduction of positive electrode materials so as to increase their sodium content. We tested this possibility using the P2-type layered oxide phase, Na_0.67_[Fe_0.5_Mn_0.5_]O_2_, which can be electrochemically reduced at a potential near 1.5 V to form the P′2-Na_1_[Fe_0.5_Mn_0.5_]O_2_ phase that delivers a capacity of ∼190 mAh g^−1^ (ref. 24). Attempts to synthesize P′2 type Na_1_[Fe_0.5_Mn_0.5_]O_2_ through direct solid-state reaction have so far failed, resulting in an O3 type phase with poor capacity^10^. Stoichiometric amounts of Na_0.67_[Fe_0.5_Mn_0.5_]O_2_ (XRD shown in Fig. 2b) and Na metal were added under argon into a hardened steel ball milling jar using a ball to powder weight ratio of 35. The XRD pattern of the obtained powder (Fig. 2a) is consistent with the reflections reported for the electrochemically produced P′2 phase^24^, which confirms the successful production of fully sodiated P′2-Na_1_[Fe_0.5_Mn_0.5_]O_2_ after only 2 h of ball milling. At shorter milling time or when a sub-stoichiometric amount of Na is used, the ball-milled samples are a mixture of the P2 and P′2 phases.

Fig. 2c–e compare the electrochemical behaviour of a Na/P2 cell with that of a Na/P′2 cell with the P′2 phase made by ball milling with Na. Note that only 0.45 Na^+^ can be removed from the P2-based cell, as opposed to nearly 0.8 Na^+^ for the P′2 cell, clearly confirming the success of the ball milling-induced sodiation process. Aside from this difference, the cells behave identically in terms of reversible and sustained capacity upon cycling, independently of whether pristine P2 or P′2 obtained by Na ball milling was initially used.

**Figure 3:**
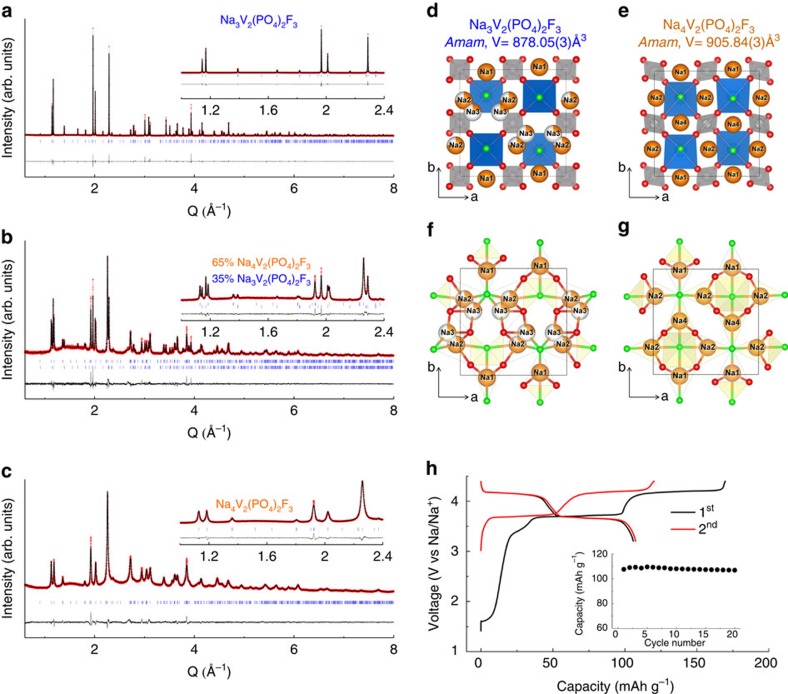
Synthesis and electrochemistry of Na_4_V_2_(PO_4_)_2_F_3_ by Na reductive ball milling. Rietveld refinements of pristine Na_3_V_2_(PO_4_)_2_F_3_ (**a**), a mixture of Na_3_V_2_(PO_4_)_2_F_3_ and Na_4_V_2_(PO_4_)_2_F_3_ (**b**) and pure Na_4_V_2_(PO_4_)_2_F_3_ (**c**). The red crosses, black continuous line and bottom grey line represent the observed, calculated and difference patterns, respectively. Vertical blue tick bars mark the Bragg reflections arising from the *Amam* space group. Patterns are given in Q-space for allowing a direct comparison. Structure of Na_3_V_2_(PO_4_)_2_F_3_ (**d**,**f**) and of Na_4_V_2_(PO_4_)_2_F_3_ (**e**,**g**) as deduced from the refinement of the synchrotron patterns. The Na environment of Na_3_V_2_(PO_4_)_2_F_3_ and Na_4_V_2_(PO_4_)_2_F_3_ is respectively highlighted in (**f**,**g**). VO_4_F_2_ octahedral and PO_4_ tetrahedral are coloured in blue and grey, respectively. Na and F atoms are shown as orange and green balls, respectively. Vacancies on the Na1 and Na2 sites on Na_3_V_2_(PO_4_)_2_F_3_ are coloured in white. The electrochemical behaviour of composite ‘Na_3.5_V_2_(PO_4_)_2_F_3_' (that is, with equal amounts of Na_3_V_2_(PO_4_)_2_F_3_ and Na_4_V_2_(PO_4_)_2_F_3_) in Na-half cell is shown in **h**.

In addition to layered oxides, the polyanionic compound NVPF (Na_3_V_2_(PO_4_)_2_F_3_) is also of great interest as a positive electrode in Na-ion cells^25^,^26^ since it shows high-voltage plateaus near 3.6 and 4.2 V, whose equal amplitudes provide a cumulative capacity of∼110 mAh g^−1^. To generalize our approach, we explored the ball milling-driven reactivity of Na against NVPF. Na_3_V_2_(PO_4_)_2_F_3_ powders were mixed with various amounts of Na and various ball milling times (Supplementary Fig. 1). To our surprise, ball milling 1 molar equivalent of NVPF with 2 equivalents of Na metal lumps for 30 min (ball to powder weight ratio of 35) results in loose composite powders whose XRD pattern differs from that of NVPF with namely the onset of extra peaks corresponding to the onset of a second phase. By increasing the milling time we progressively increase the amount of this extra phase, which was obtained as a single phase after 3 h of ball milling (Fig. 3a–c). The elemental distribution and chemical composition of this as-prepared powder was studied by high-angle annular dark field scanning transmission electron microscopy. The images demonstrate that crystallites of the main phase are surrounded by Na nanoparticles with sizes of 20–50 nm, as deduced from compositional energy-dispersive X-ray spectroscopy mapping (Supplementary Fig. 2a,b). Note that this is in agreement with the residual amount of Na metal, as deduced by differential scanning calorimetry (DSC) (Supplementary Fig. 3). Moreover, the analysis of the main phase crystallites provides a Na:V:P=4.0(2):2.0(1):2.1(2) atomic ratio, consistent with a Na_4_V_2_(PO_4_)_2_F_3_ formulae.

The Synchrotron XRD patterns of the pristine NVPF and Na_4_V_2_(PO_4_)_2_F_3_ phase, and of a mixture of both phases are shown in Fig. 3a–c. The diffraction peaks of the phase formed upon ball milling with Na can be indexed in the same orthorhombic cell as for the pristine NVPF^26^, but with different lattice parameters, that is, *a*=9.2208(2) Å, *b*=9.2641(2) Å and *c*=10.6036(2) Å. This corresponds to an increase of the unit cell by 3.2% (*V*=905.79(3) Å^3^) relative to the pristine NVPF phase (*V*=878.05(3) Å^3^), which is consistent with the uptake of extra sodium upon reduction.

Refinement of the synchrotron XRD pattern (Fig. 3c) of the obtained Na_4_V_2_(PO_4_)_2_F_3_ was undertaken based on the structural model reported for pristine NVPF by Bianchini *et al*.^26^ The VO_6_ octahedra and PO_4_ tetrahedra arrangement of NVPF is kept, and the best agreement between the observed and calculated patterns was found for the Na positions as listed in Supplementary Tables 1 and 2 and Supplementary Note 1. The Na environments are shown in Fig. 3e. The three distinct Na sites are all seven-fold coordinated with four oxygen and three fluorine atoms, which is analogous to the coordination of Na1 in NVPF (Fig. 3g). The structural analysis fully confirms the chemical composition (Na_4_V_2_(PO_4_)_2_F_3_) and indicates that there is apparently no further space for Na insertion. Lastly, the synchrotron XRD patterns of the sample prepared by ball milling NVPF with Na for 30 min can be perfectly refined with a two phases model: NVPF and Na_4_V_2_(PO_4_)_2_F_3_, as shown in Fig. 3b.

The occurrence of Na_4_V_2_(PO_4_)_2_F_3_ comes as a total surprise, as no extra capacity has ever been reported for NVPF at low potential. It is however worth noting that the reversible insertion of 1 Na^+^ at 0.3 V was just recently reported for Na_3_V_2_(PO_4_)_3_ (ref. 27). Knowing the existence of the two Na_3_V_2_(PO_4_)_2_F_3_ and Na_4_V_2_(PO_4_)_2_F_3_ end-member phases, we deliberately prepared composites of nominal compositions having different amounts of NVPF and Na_4_V_2_(PO_4_)_2_F_3_ that will be denoted hereafter ‘Na_3+*x*_V_2_(PO_4_)_2_F_3_'. Such as-prepared composites show similar electrochemical performance as NVPF, except *x* more Na is removed during the first oxidation (Fig. 3h). They deliver a stable capacity of ∼110 mAh g^−1^ when cycled between 4.4 and 3 V.

**Figure 4:**
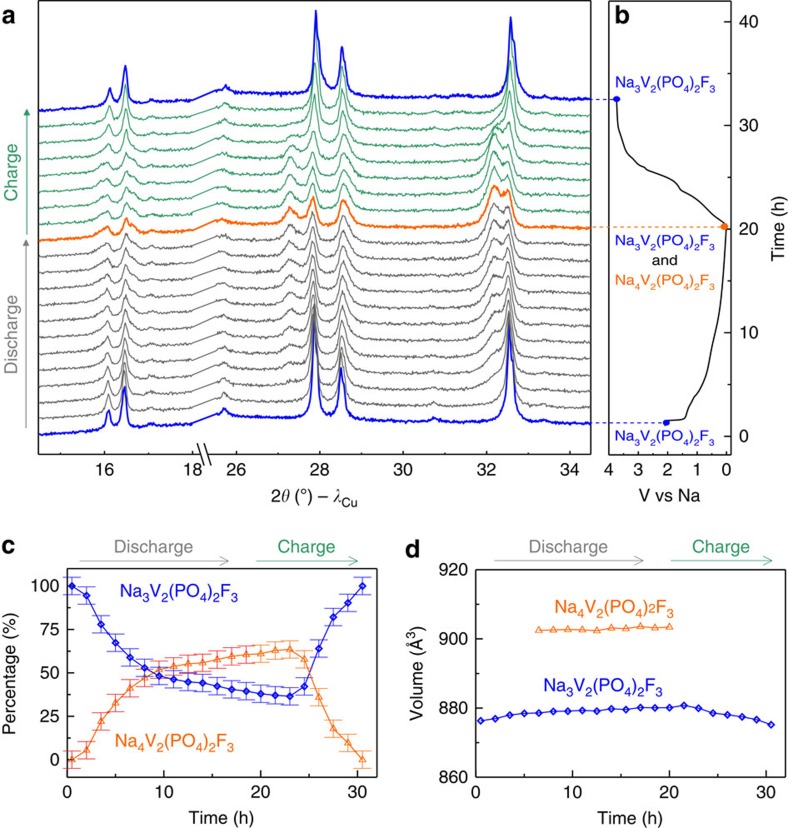
*In situ* XRD patterns collected for a Na_3_V_2_(PO_4_)_2_F_3_/Na cell. XRD patterns (**a**) and the corresponding electrochemical curve (**b**) is shown. The percentage of each phase and their unit cell volumes are shown in **c**,**d**, respectively. Error bars represent standard deviation obtained from the Rietveld refinement. Note that the Na_4_V_2_(PO_4_)_2_F_3_ phase mainly forms at the beginning of the process (within the 1.4–0.8 V domain) and then the Na-uptake appears self-limiting for reasons explained with the text. For time >20 h the amount of Na_4_V_2_(PO_4_)_2_F_3_ within the accuracy of the measurements still increases, while the cell is already in its oxidation mode.

In light of our finding on the Na_4_V_2_(PO_4_)_2_F_3_ phase, we explored electrochemical intercalation in NVPF down to low voltages. *In situ* XRD measurements were conducted on a Na/NVPF cell, with XRD patterns collected for every 90 min. As the cell was being discharged at 0.15 C, we observed the progressive appearance of an additional set of peaks (Fig. 4a,b) corresponding to the Na_4_V_2_(PO_4_)_2_F_3_ phase obtained by ball milling. They appear at the expense of the NVPF reflections, which barely change in position but decrease in intensity. By careful refinement of the XRD patterns, we quantified (Fig. 4c) the growing amounts of Na_4_V_2_(PO_4_)_2_F_3_, promoted by continuously lowering the reduction voltage; Na_4_V_2_(PO_4_)_2_F_3_ constitutes nearly 60% of the composite when the cell potential reaches 0 V. This corresponds to a *x* value of 0.6 (in ‘Na_3+*x*_V_2_(PO_4_)_2_F_3_'), which cannot be determined accurately from coulometric titration because of side reactions (Supplementary Note 2 and Supplementary Figs 4 and 5). Turning to the charging process, a reverse trend is observed, with the recovery at 3.6 V of an XRD pattern similar to that of the pristine phase indicating the full reversibility of the Na-uptake–removal process (Fig. 4a). However, it is worth noting the drastic difference in the charge and discharge profiles, highlighted in the derivative plot (Supplementary Fig. 4a,b), which is indicative of a different reacting pathway. Moreover, we note the continuous growing of Na_4_V_2_(PO_4_)_2_F_3_ when the cell is switched back to oxidation, further implying a complex Na-uptake–removal process. We believe this partial reduction to be nested in kinetics blockages that are most likely due to the growth of a thick insulating SEI layer due to copious electrolyte decomposition and/or the formation of peculiar self-limiting core−shell-like ‘Na_3+*x*_V_2_(PO_4_)_2_F_3_' particles upon reduction. Besides, attempts to modify the SEI with the use of fluoroethylene carbonate (FEC) did not result in subsequent changes in increasing the amount of the Na_4_V_2_(PO_4_)_2_F_3_ phase (Supplementary Note 2). In comparison, there are two specific aspects that facilitate the production of pure Na_4_V_2_(PO_4_)_2_F_3_ in the case of ball milling synthesis. They enlist the absence of SEI formation because of the lack of electrolyte, and the continuous formation of highly reacting fresh surfaces due to repeated fracturing.

**Figure 5:**
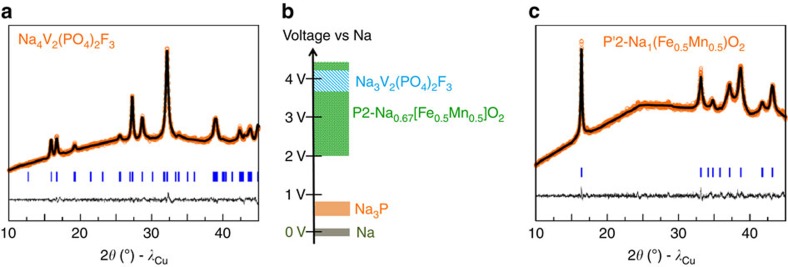
Synthesis of Na_4_V_2_(PO_4_)_2_F_3_ and P′2 phase by Na_3_P ball milling. The XRD Rietveld refinement of (**a**) Na_4_V_2_(PO_4_)_2_F_3_ and the profile matching of (**c**) P′2-Na_1_[Fe_0.5_Mn_0.5_]O_2_ phase obtained by ball milling using Na_3_P as a reducing reagent are shown together with in the middle an electrochemical scale ranking the various used compounds in terms of redox potentials (**b**). In **a**,**c** the orange open circles, black continuous line and bottom grey line represent the observed, calculated, and difference patterns, respectively. Vertical blue tick bars mark the Bragg reflections.

These examples highlight the benefits of ball milling-driven Na-reduction reactions, which are free of the complexities associated with handling reactive solutions or using mild temperature processing to prepare fully reduced materials. The synthesized P′2-Na_1_[Fe_0.5_Mn_0.5_]O_2_ and ‘Na_3+*x*_V_2_(PO_4_)_2_F_3_' show similar cyclic and rate performance (Supplementary Fig. 6) as the pristine P2-Na_0.67_[Fe_0.5_Mn_0.5_]O_2_ and NVPF, respectively, demonstrating that the short ball milling time does not bring any detrimental effect to its electrochemical performance. Such reactions, leading to the transformation of P2 to P′2, or NVPF to Na_4_V_2_(PO_4_)_2_F_3_, are topotatic since the host structural frameworks are unaltered, and can simply be viewed as insertion reactions. Thus, the reactivity is simply dictated by the redox potential associated with the insertion of Na in various compounds. As shown in the electrochemical energy scale schematic representation in Fig. 5b, although Na_3_P is a milder reducing reagent than Na (0.5 V versus Na^+^/Na^0^), it is also expected to reduce P2 and NVPF. To test this point, as we have done for Na, a survey of various ball milling times together with various amounts of Na_3_P were conducted and the obtained composites were periodically checked for phase purity. We find the possibility to successfully prepare the Na_4_V_2_(PO_4_)_2_F_3_ phase (Fig. 5a) as well as the P′2 phase (Fig. 5c) by ball milling powdered mixtures of NVPF and P2 with 1 and 0.2 molar amounts of Na_3_P, respectively. The reaction between Na_3_P and NVPF could be classified as chemical sodiation. The successful sodiation of NVPF by Na_3_P suggests that the reduction potential of NVPF should be higher than the potential for Na_3_P oxidation, that is, ∼0.5 V. The advantage of Na_3_P as compared with Na metal is the fact it is a powder, although there is a compromise for this convenience with the requirements of excess amounts of Na_3_P and longer ball milling time.

### Na-enriched phases for highly efficient Na-ion batteries

**Figure 6:**
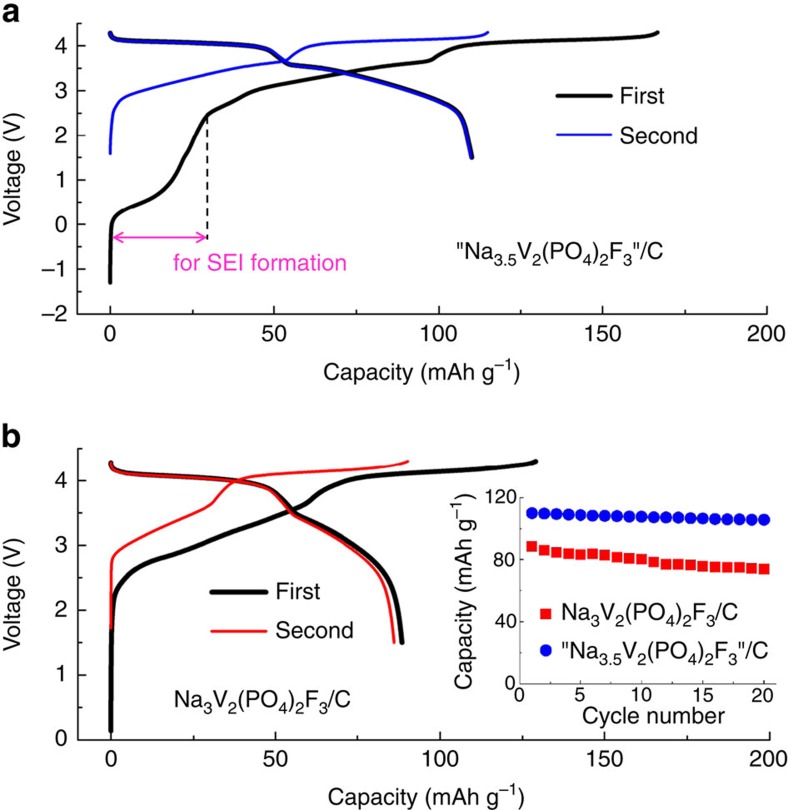
Performances of C/‘Na_3+*x*_V_2_(PO_4_)_2_F_3_' Na-ion cells. The voltage-composition profiles are reported for two first cycles for cell C1 using Na_3_V_2_(PO_4_)_2_F_3_ (**b**) and cell C2 using ‘Na_3.5_V_2_(PO_4_)_2_F_3_' (**a**). The weight ratio of cathode to anode is 2.7 and 1.9 for C1 and C2 cell, respectively. The cells were cycled at a 0.2C rate (C rate being defined as the extraction of 2 Na from NVPF). For both cells a 1 M NaClO_4_ in EC/DMC (50/50 by volume) electrolyte was used. The capacity is calculated based on the weight of positive materials. Inset in (**b**) shows the capacity retention in the first 20 cycles.

The successful preparation of Na-rich Na_4_V_2_(PO_4_)_2_F_3_ or composites with known amounts of NVPF and Na_4_V_2_(PO_4_)_2_F_3_ enables control over the extra Na content by playing with ball milling times, and is of great importance for enhancing the performances of C/NVPF Na-ion cells. This situation closely mirrors the use of Li_1+*x*_Mn_2_O_4_ as a Li source in C/LiMn_2_O_4_ Li-ion cells^12^, since the extra Na^+^ ions present in NVPF can be removed at low potential without an added weight penalty with exception to the weight of the *x* added Na. As a proof of concept, we assembled various electrochemical cells having carbon as the negative electrode and either the pristine NVPF phase or the ‘Na_3+*x*_V_2_(PO_4_)_2_F_3_' composites as the positive electrode. For conciseness, we focus here only on the performance for the optimum *x* value. This value was estimated to be equal to 0.5 for compensating the ∼25% irreversible capacity related to the SEI. Details about the balancing of cathode and anode are described in Supplementary Fig. 7 and Supplementary Note 3. Figure 6 shows the electrochemical performances of cells C1 and C2, having pristine NVPF and composite ‘Na_3.5_V_2_(PO_4_)_2_F_3_' as positive electrodes, respectively. The cells were tested electrochemically between 1.5 and 4.3 V. The voltage trace for C1 mirrors reports in the literature for similar cells with a charging capacity of 129 mAh g^−1^ and a discharge capacity of 89 mAh g^−1^, which remains stable upon cycling. Providing sacrificial Na in the form of Na-rich NVPF (C2) strongly modifies the voltage profile: an initial capacity near 0.5 V corresponds to the removal of Na from ‘Na_3.5_V_2_(PO_4_)_2_F_3_' to compensate for the SEI formation at the negative electrode; afterwards the potential rises, associated with removal of Na from NVPF. The C2 cell exhibits an overall charging capacity of 167 mAh g^−1^ and a discharge capacity of 110 mAh g^−1^, which is a 24% enhancement compared with cell C1. This corresponds to an overall 10% increase in energy density as described in Supplementary Note 4. Lastly, there is no evidence to suggest that use of the Na-rich phase jeopardizes the cycle life, with the capacity remaining nearly constant over 20 cycles (the maximum we have tried). Needless to say that further optimization of cell balancing via the use of three electrodes is being pursued to fine tune the proper value of *x* for achieving optimum performance.

We next implemented a similar approach in the optimization of C/P2-type Na_0.67_[Fe_0.5_Mn_0.5_]O_2_ Na-ion systems. In contrast to NVPF, the positive P′2-Na_1_[Fe_0.5_Mn_0.5_]O_2_ phase cannot act as an extra source of Na since Na-rich Na_1+*x*_[Fe_0.5_Mn_0.5_]O_2_ does not exist. This is easily overcome by first preparing the P′2-Na_1_[Fe_0.5_Mn_0.5_]O_2_ phase by Na-ball milling and then homogeneously mixing the P′2 phase with the proper amount of Na_3_P to compensate for the carbon irreversible capacity. We here use extra Na_3_P, rather than Na metal, as it is easily added as powders to positive electrode materials to make homogeneous composite electrodes. Upon charging the cell, the added Na_3_P will oxidize to compensate for the Na consumed in SEI formation during the first cycle. This leaves behind elemental phosphorous, which remains as an electrochemical spectator within the cell upon subsequent cycles (Supplementary Fig. 8), but which also has the capability to act as a safety buffer by reinserting Na at a constant voltage in case of cell over-discharge.

**Figure 7:**
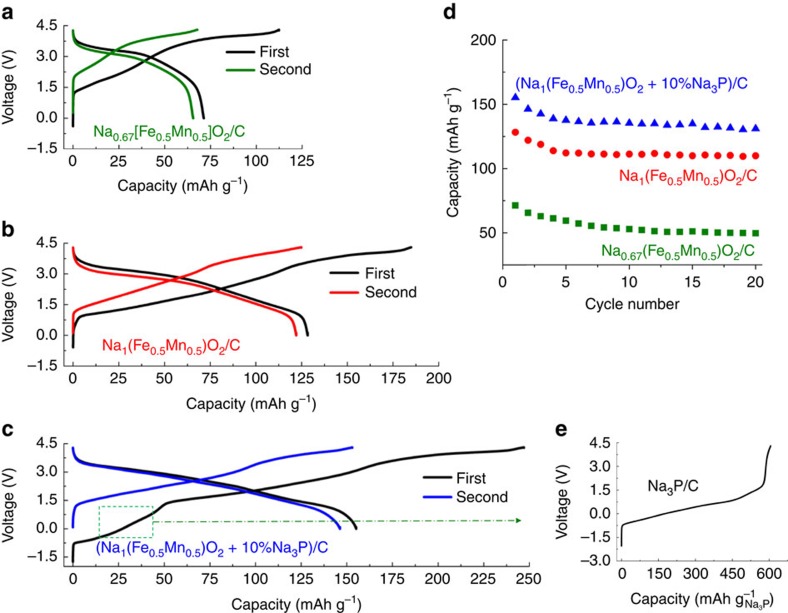
Performances of C/P2-Na_0.67_[Fe_0.5_Mn_0.5_]O_2_ Na-ion cells. The voltage-composition profiles are reported for the two first cycles for cells using (**a**) as-made P2-Na_0.67_[Fe_0.5_Mn_0.5_]O_2_ (cell D1), (**b**) P′2-Na_1_[Fe_0.5_Mn_0.5_]O_2_ made by Na ball milling (cell D2) and (**c**) for a P′2-Na_1_[Fe_0.5_Mn_0.5_]O_2_/Na_3_P composite (cell D3) as positive electrode. The negative electrode is C. The weight ratio of positive to negative electrode is 2.6, 1.6 and 1.4 for D1, D2 and D3 cell, respectively. The cells were cycled at 0.1 C rate. The capacity is based on the weight of positive materials, including Na_3_P for D3 cell. The capacity retention of D1, D2 and D3 cell is given (**d**), and **e** shows the first charge profile of a C/Na_3_P cell. For all cells a 1 M NaClO_4_ in EC/DMC (50/50 by volume) electrolyte was used.

A series of full Na-ion cells were assembled using carbon negative electrodes and composite positive electrodes made of P2-Na_0.67_[Fe_0.5_Mn_0.5_]O_2_ (cell D1), P′2-Na_1_[Fe_0.5_Mn_0.5_]O_2_ (cell D2) and P′2-Na_1_[Fe_0.5_Mn_0.5_]O_2_ +10 wt.% Na_3_P (cell D3). The corresponding voltage profiles for the cells, collected upon cycling between 0 and 4.3 V at a current rate of 0.1C, are shown in Fig. 7a–c. The D1 cell shows a charge capacity of 112 mAh g^−1^ and a discharge capacity of only 71 mAh g^−1^ that is maintained upon subsequent cycling. These capacities are considerably lower than those obtained for Na/Na_0.67_[Fe_0.5_Mn_0.5_]O_2_ half cells (168 mAh g^−1^), due to the replacement of the Na anode by carbon, and hence the absence of a Na source to enable formation of the P′2 phase and to compensate for losses to the SEI at the carbon electrode. In contrast, the D2 and D3 cells show charge capacities of 185 and 247 mAh g^−1^, respectively, with corresponding discharge capacities of 128 and 155 mAh g^−1^ that stabilized to 110 and 131 mAh g^−1^ after 20 cycles (Fig. 7d), the maximum we have tried so far. Note that cell D3, as opposed to cell D2, presents an extra voltage feature below 1 V in the first charge. The feature mirrors the voltage charge profile of a Na_3_P/C cell (Fig. 7e), which nicely demonstrates the way Na_3_P works as a sacrificial Na source in a full Na-ion cell. Owing to its 0.5 V redox potential versus Na^+^/Na^0^, Na_3_P initially clamps the positive electrode voltage at a lower potential than that of the negative electrode-carbon starts to uptake Na^+^ at solely 1.5 V versus Na^+^/Na, thus resulting in a negative output voltage of the cell to start with. Once the cell is charged, Na^+^ ions are released from Na_3_P to compensate for the irreversible SEI formation on carbon with the cell voltage climbing to 1 V; afterwards Na^+^ is released from the P′2 part of the positive electrode so that the voltage profiles of both cells become nearly identical. Application wise the observed increase in reversible capacities from 71 to 128 and 155 mAh g^−1^ for the D1, D2, and D3 cells, respectively, which occurs without any sacrifice to the capacity retention behaviour upon cycling (Fig. 7d), clearly demonstrates the benefits of pre-producing the P′2 phase and incorporating Na_3_P as a Na reservoir. Such capacity improvements increase the energy density by 30% between C/P2- Na_0.67_[Fe_0.5_Mn_0.5_]O_2_ and C/P′2-Na_1_[Fe_0.5_Mn_0.5_]O_2_ Na-ion cells, and by an additional of 7% using P′2-Na_1_[Fe_0.5_Mn_0.5_]O_2_/Na_3_P composites rather than solely P′2-Na_1_[Fe_0.5_Mn_0.5_]O_2_ powders as positive electrodes (Supplementary Note 4).

## Discussion

We have reported the preparation of Na-based alloys (Na_3_Sb, Na_3_P and so on) via an approach that relies on the ball milling of Na metal and have demonstrated that such an approach, using either Na or Na_3_P as reducing agents, can equally be used to easily prepare P′2-Na_1_[Fe_0.5_Mn_0.5_]O_2_ layered oxide insertion compound, and to produce ‘Na_3+*x*_V_2_(PO_4_)_2_F_3_' composites with the *x*=1 composition being a single phase never reported so far. Such findings provide new insights for combating the irreversible capacity of NIBs, thereby significantly enhancing their performances. We demonstrate the feasibility of assembling full Na-ions cells showing marked enhancements in energy storage density (10–30%) by using ‘Na_3+*x*_V_2_(PO_4_)_2_F_3_' and P′2-Na_1_[Fe_0.5_Mn_0.5_]O_2_ as positive electrodes, respectively, with an extra 7% been achievable for the latter by adding proper amounts of Na_3_P sacrificial salt to P′2-Na_1_[Fe_0.5_Mn_0.5_]O_2_ powders. The improvement in energy density associated to the addition of Na_3_P is here limited due to the low voltage of cathode at the end of the full discharge of the Na-ion cell. Since *N*-methyl-2-pyrrolidone (NMP) is used in today's electrode formulation technology we have checked the reactivity of Na_3_P towards NMP by directly immersing Na_3_P/C composite in NMP overnight. No change has been observed in the Na_3_P crystallinity and its electrochemical activity was preserved with a capacity of ∼600 mAh g^−1^ when oxidized to 4.3 V. Nevertheless, an inherent difficulty, application wise, with such fully sodiated electrodes is their reactivity towards moisture, hence the need to design coating-grafting techniques to minimize such moisture sensitivity^28^ as it is being eagerly pursued in our group. Despite this practical pending issue, we anticipate that the here discussed means for enhancing the performances of NIBs will have strong implications towards their upcoming commercialization.

## Methods

### Synthesis of Na_3_P

Stoichiometric amounts of metallic sodium as bulk (Sigma) and red phosphorus (Alfa, 325 mesh) were filled into a hard steel ball-milled jar (30 cm^3^) of a Spex 8000M ball-miller in an Ar-filled glove box and equipped with seven hard steel balls each having a weight of 7 g and a diameter of 12 mm. These solid materials were ball-milled for 2 h to obtain Na_3_P particles. The mass ratio of balls to Na_3_P was maintained at 35. A similar protocol was used to prepare the reported Na_3_Sb powders.

### Synthesis of P′2 type Na_1_[Fe_0.5_Mn_0.5_]O_2_

P2 phase of Na_0.67_[Fe_0.5_Mn_0.5_]O_2_ was produced by solid-state reaction under 900 °C for 12 h in air^10^. It was ball milled with stoichiometric amount of Na to produce Na_1_[Fe_0.5_Mn_0.5_]O_2_ for durations from 20 min to 2 h. Once the obtained powders were single phased, 20 wt% carbon SP was added and the mixture was ball milled for additional 10 min for making electrodes. Excess amounts of Na_3_P (0.2 mole Na_3_P per Na_0.67_[Fe_0.5_Mn_0.5_]O_2_) was also used to transfer P2 into P′2 phase without noting any detrimental effect on the phase formation.

### Synthesis of ‘Na_3+*x*_V_2_(PO_4_)_2_F_3_' composites

Pure NVPF was obtained from CEA via a recently patented process. It was ball milled with increasing amounts of Na ranging from 0.5 Na to 2 Na per mole of NVPF and times ranging from 20 min to 3 h. We find the formation of single phased materials for a stoichiometry of 2 Na and for ball milling time >3 h (Supplementary Fig. 1). Such samples were shown to contain tiny amounts of remaining Na as deduced by DSC experiments (Supplementary Fig. 3). Equally, we could produce the fully sodiated Na_4_V_2_(PO_4_)_2_F_3_ phase by ball milling for 3 h of NVPF with 1 M of Na_3_P. Composites with adjusted values of *x* in ‘Na_3+*x*_V_2_(PO_4_)_2_F_3_' to compensate for the carbon SEI negative electrode were made as above by properly adjusting amount of Na and ball milling time. Once the desired composite obtained, it was ball milled for 10 min with 20% additional carbon SP to make the electrode.

### X-ray diffraction

XRD patterns were collected on a Bruker D8-Advance diffractometer equipped with Cu Kα radiation source. Additional synchrotron XRD patterns were collected on powders put in sealed glass capillaries (diameter 0.7 mm) either at the European Synchrotron Radiation Facility on ID22 with *λ*=0.3543 Å (Fig. 3a,c) or at 11BM-Argonne National Lab with *λ*=0.4142 Å (Fig. 3b). The *in situ* XRD patterns were recorded using electrochemical cells, assembled similarly to our Swagelok cell, but equipped with a beryllium window as current collector on the positive side. These cells were placed on the Bruker D8-Advance diffractometer (Cu Kα radiation) and connected to the VMP2 system. All patterns were analysed using the Rietveld method as implemented in the FullProf program^29^. Phase quantification was performed on the *in situ* patterns by applying a overall correction on the patterns to account for the absorption from the Be window.

### Electrochemical tests

Coin cells were used to study the electrochemical performances. 1 M NaClO_4_ solution in a mixture of EC/DMC in 1:1 ratio was used as electrolyte for the full cells with C as negative electrode. 5% FEC was systematically added in half cells when Na metal was used as counter electrode. The only exception regards the cells made of NVPF as positive electrode that were initially discharged because of experimented interferences between the decomposition potential of FEC and the insertion reduction potential of Na into NVPF (Supplementary Figs 4 and 5). Two pieces of Whatman glass fibres soaked with the electrolyte were used as separator between the positive and negative electrode. The powders of active materials were mixed with 20% carbon SP using Spex 8000M mixer mill. A typical weight of 5 mg of active electrode material was used per cell whatever Swagelok's or coin cells. The cells were galvanostatic charged/discharged with a VMP automatic cycling/data recording system (Biologic Co., Claix, France) using various ranges of scanning potential and C rates with 1 C corresponding to the uptake or removal of 1 Na^+^ and 2 Na^+^ per formula unit in 1 h for P2- Na_0.67_[Fe_0.5_Mn_0.5_]O_2_ and NVPF, respectively. Note: While the present manuscript was under review, the Na_4_V_2_(PO_4_)_2_F_3_ phase was predicted, but not synthesized, through computational calculation by Ceder's group^29^.

## Additional information

**How to cite this article:** Zhang, B. *et al*. Insertion compounds and composites made by ball milling for advanced sodium-ion batteries. *Nat. Commun.* 7:10308 doi: 10.1038/ncomms10308 (2016).

## Supplementary Material

Supplementary Information Supplementary Figures 1-8, Supplementary Tables 1-2 and Supplementary Notes 1-4
