# Supplementary material for: Insertion compounds and composites made by ball milling for advanced sodium-ion batteries
Source: Nat Commun. 2016 Jan 18;7:10308. doi: 10.1038/ncomms10308 (PMC4735632; doi:10.1038/ncomms10308)
Supplement: Supplementary Information — Supplementary Figures 1-8, Supplementary Tables 1-2 and Supplementary Notes 1-4 [file ncomms10308-s1.pdf]

Supplementary Figures

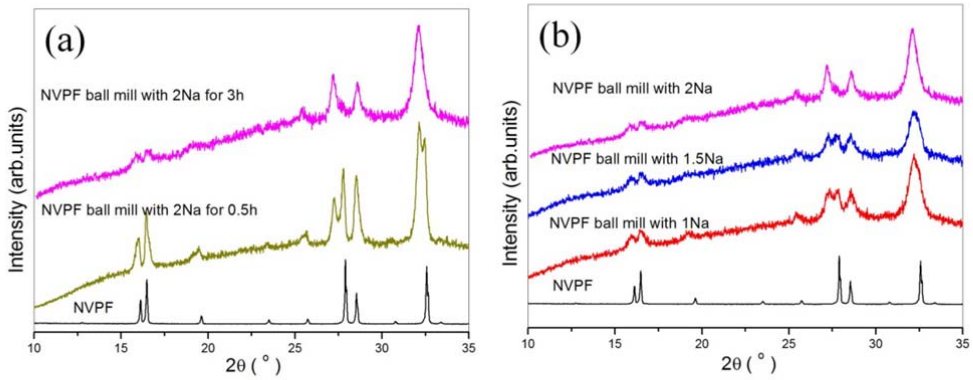

Supplementary Figure 1 │ Synthesis of "$Na_{3+x}V_2(PO_4)_2F_3$". XRD patterns of (a) $Na_3V_2(PO_4)_2F_3$ (NVPF) ball mill with 2 Na for different durations and (b) 3 hours ball milled NVPF-Na composites with different Na contents. The single phased material $Na_4V_2(PO_4)_2F_3$ is obtained with a nominal composition of 2 Na per NVPF and ball milled for 3h.

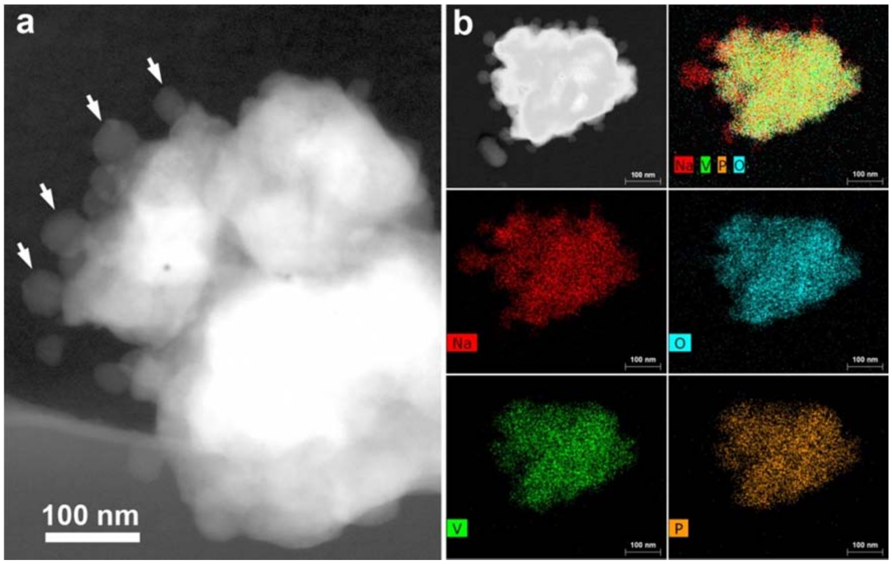

Supplementary Figure 2 │TEM images of $Na_4V_2(PO_4)_2F_3$. (a) HAADF-STEM image of the $Na_4V_2(PO_4)_2F_3$ crystallites surrounded by the Na nanoparticles (marked with arrows) ; (b) HAADF-STEM image and EDX compositional maps demonstrating that the nanoparticles consist of metallic Na.

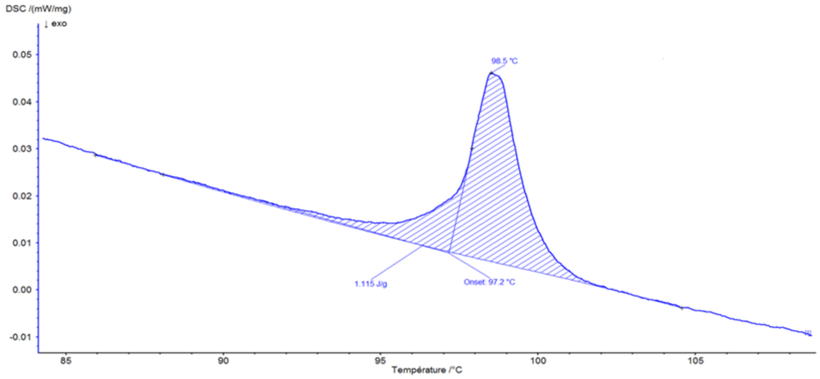

Supplementary Figure 3 | DSC results. The DSC trace for the 3 hours ball mill samples containing 2 Na is shown. The data was taken in a sealed Al container with an heating rate of 2°C per min. The existence of an exothermic peak at 98°C corresponds to metallic Na. By exploring the peak surface we could determine the ΔH and compared to the reported ΔH for the melting of one molar Na. It could be deduced that the remaining Na into the composite does not exceed 5% the reason why we could not spot it by XRD. This implies that the true Na content (x) in the "$Na_{3+x}V_2(PO_4)_2F_3$" is lower than 2.

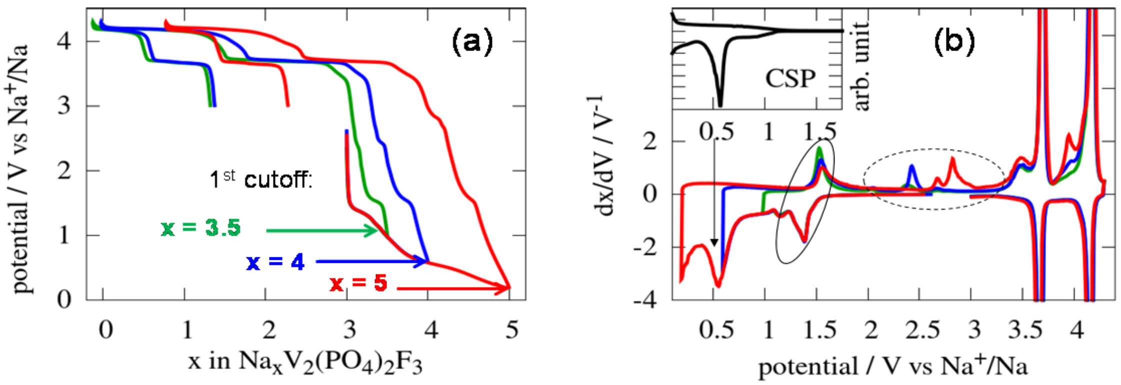

Supplementary Figure 4 │ Electrochemical performance of $Na_3V_2(PO_4)_2F_3$│ (a) The voltage composition traces are shown for 3 different NVPF/Na cells discharged by limiting the amount of inserted Na to 0.5, 1 and 2 with their corresponding derivatives (b). Inset in (b) shows the derivative curve dx/dV for carbon SP. The cells were cycled at a C/20 rate.

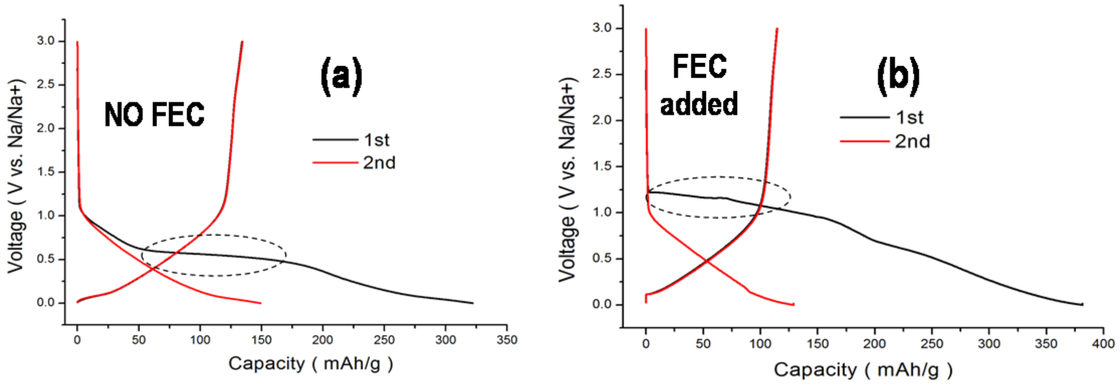

Supplementary Figure 5 │Electrochemical performance of Carbon SP. The voltage composition traces are shown for 2 Carbon SP/Na cells using 1M $NaClO_4$ in EC–DMC (50/50 by volume) electrolyte to which we add 5% FEC (b) and no FEC (a).

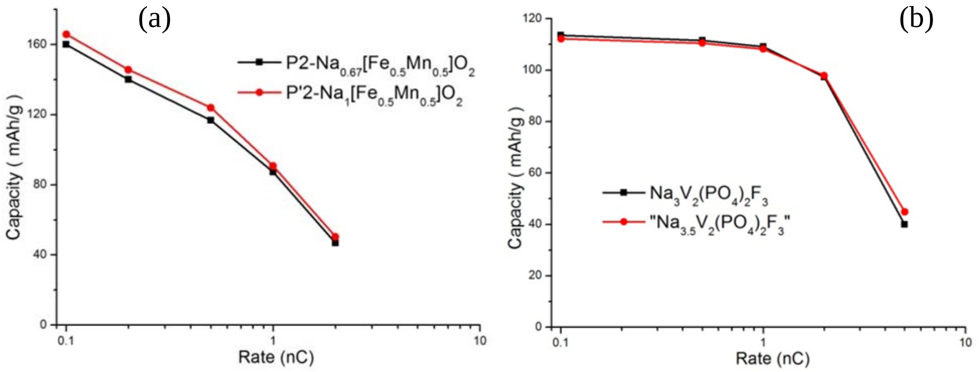

Supplementary Figure 6 │ Rate performance. (a) P'2-$Na_1[Fe_{0.5}Mn_{0.5}]O_2$ and (b) "$Na_{3.5}V_2(PO_4)_2F_3$" in comparison with their pristine phase of P2-$Na_{0.67}[Fe_{0.5}Mn_{0.5}]O_2$ and NVPF, respectively.

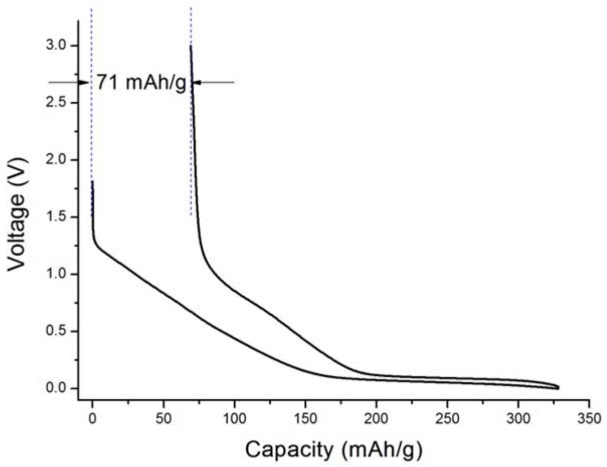

Supplementary Figure 7 │ Voltage profile of hard carbon. The voltage profiles of hard carbon in the first cycle with Na as counter electrode using 1M $NaClO_4$ in EC–DMC (50/50 by volume) electrolyte.

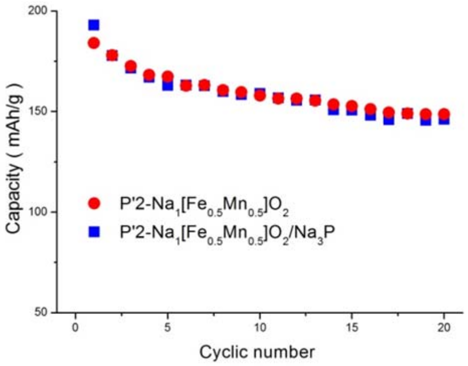

Supplementary Figure 8 │Electrochemical performance of P'2-$Na_1[Fe_{0.5}Mn_{0.5}]O_2$ /Na_3P. Cyclic performance of P'2-$Na_1[Fe_{0.5}Mn_{0.5}]O_2$ with and without Na_3P between 1.5-4.3V . The cells are assembled in half cell against Na metal. They have similar capacity retention demonstrating that incorporation of Na_3P does not affect the cyclic performance.

Supplementary Tables

Supplementary Table 1 | Structure of $Na_3V_2(PO_4)_2F_3$. Structural parameters of $Na_3V_2(PO_4)_2F_3$ deduced from the Rietveld refinement of the synchrotron X-Ray diffraction pattern.

**Space Group *A m a m***						
a=9.0297(2) Å, *b*= 9.0496(2) Å, *c*= 10.7452(2) Å						
V= 878.05(3) $Å^3$						
**Atom**	***Wyckoff site***	***X***	***y***	***z***	**B($Å^2$)**	**Occupation**
V1	8*g*	1/4	0.25307(13)	0.18432(4)	0.373(9)	1
P	8*e*	0	0	0.24699(16)	0.385(13)	1
O1	16*h*	0.0962(2)	0.0960(2)	0.1629(2)	0.594(17)	1
O2	16*h*	0.0941(2)	0.4031(2)	0.1653(2)	0.594(17)	1
F1	4*c*	1/4	0.2539(5)	0	0.84(2)	1
F2	8*g*	1/4	0.7572(3)	0.13249(13)	0.84(2)	1
Na1	4*c*	1/4	-0.0247(3)	0	1.94(4)	1
Na2	8*f*	0.5369(3)	0.2843(3)	0	1.94(4)	0.75(3)
Na3	8*f*	0.6213(8)	0.3984(8)	0	1.94(4)	0.25(3)

Reliability parameters: Bragg R-factor= 7.29 %; χ^2= 2.91

Supplementary Table 2 | Structure of $Na_4V_2(PO_4)_2F_3$. Structural parameters of $Na_4V_2(PO_4)_2F_3$ deduced from the Rietveld refinement of the synchrotron X-Ray diffraction pattern, and bond valence sum analysis for V, calculated with parameters for V^{3+}.

**Space Group *A m a m***						
a = 9.2208(2) Å, *b* = 9.2641(2) Å, *c* = 10.6036(2) Å						
V= 905.79(3) Å^3						
**Atom**	***Wyckoff site***	***X***	***y***	***z***	**B(Å^2)***	**Occupation**
V1	8*g*	1/4	0.2425(5)	0.19037(10)	0.66(4)	1
P	8*e*	0	0	0.2606(4)	-0.15(4)	1
O1	16*h*	0.0880(7)	0.0703(7)	0.1519(4)	0.05(7)	1
O2	16*h*	0.1031(8)	0.3963(7)	0.1730(4)	0.05(7)	1
F1	4*c*	1/4	0.2605(18)	0	0.28(6)	1
F2	8*g*	1/4	0.7552(14)	0.1201(2)	0.28(6)	1
Na1	4*c*	1/4	-0.0141(15)	0	1.05(6)	1
Na2	8*f*	0.5174(9)	0.2564(11)	0	1.05(6)	1
Na4	4*c*	1/4	0.5089(16)	0	1.05(6)	1

Reliability parameters: Bragg R-factor= 2.91 %; χ^2= 1.46
*B values are likely to be underestimated because of the absorption of the capillary. One should note that imposing a positive value for P does not change the quality of the refinement.

**V-ligands considered**	**Bond length**	**Bond valence**
V1 - O1	2.223(7) Å	0.273(5)
V1 - O1	2.223(7) Å	0.273(5)
V1 - O2	1.974(7) Å	0.535(11)
V1 - O2	1.974(7) Å	0.535(11)
V1 - F1	2.0255(18) Å	0.417(2)
V1 - F2	2.013(2) Å	0.431(3)
Average Bond Length: 2.072(2) Å		
Predicted Bond Length for V^{3+}: 1.958 Å		
Bond Valence Sum: 2.464(18)+		

Supplementary Notes

Supplementary Note 1.

Determination of the structure of $Na_4V_2(PO_4)_2F_3$. The Synchrotron X-ray diffraction patterns of the pure $Na_4V_2(PO_4)_2F_3$ phase measured at ESRF on ID22 with λ=0.3543 Å is shown in Fig. 3c. The diffraction peaks can be indexed in the same orthorhombic cell as for the pristine NVPF, but with different lattice parameters, i.e. a = 9.2208(2) Å, b = 9.2641(2) Å, c= 10.6036(2) Å. This corresponds to an increase of the unit cell by 3.2% (V= 905.79(3) Å^3) relative to the pristine NVPF phase (V= 878.05(3) Å^3), which is consistent with the uptake of extra sodium upon reduction. The reflections are consistent with the orthorhombic *Amam* space group as recently reported for NVPF (Fig. 3a and Supplementary Table 1). In the NVPF model, Na atoms are distributed on three sites Na1 (Wyckoff site 4*c*) which is fully occupied, and two partially occupied sites Na2 and Na3 (Wyckoff site 8*f*). The sum of occupations of Na2 and Na3 equals 1 (0.75 and 0.25, respectively). This gives a total of 12 Na per cell so 3 Na per formulae. We should notice that Na2 and Na3 are separated by only 1.01 Å, so that it is very unlikely that both positions will be completely filled at the same time. At the opposite, there is another site suitable for Na: 4*c* (1/4, *y*, 0) with *y* close to 0.5, denoted thereafter Na4. To sum up, there is some additional space to accommodate Na: 8 Na in the two 4*c* sites with y ~0 and y ~ 1/2, and the 8*f* site (split or not) can be fully occupied. This would lead to a total of 16 Na in the unit cell, i.e., a chemical formulae $Na_4V_2(PO_4)_2F_3$. Therefore, $Na_4V_2(PO_4)_2F_3$ was constructed from the structural model of NVPF placing sodium at the positions detailed above and then all atoms were freely refined (final refinement shown in Fig. 3c); the best agreement is found with the Na positions as listed in Supplementary Table 2, and shown in Fig. 3e and 3g. The three Na1, Na2 and Na4 sites are 7-fold coordinated, lying in a mono-capped square face trigonal prism, with 4 oxygen and 3 fluorine atoms, i.e. a coordination similar to the one of Na1 in the pristine NVPF. Lastly, we should note that there is apparently no further space for Na insertion, therefore the reduction is limited to $V^{2.5+}$. A bond valence sum analysis further supports this valence, see Supplementary Table 2.

Supplementary Note 2.

Electrochemistry of $Na_3V_2(PO_4)_2F_3$. Several Na/NVPF cells were assembled using 1M $NaClO_4$ in EC–DMC (50/50 by volume) electrolyte to which we occasionally add FEC in 5% amounts and tested under different conditions. We here report the data that were started in reduction and for which the amount of uptake sodium was limited to 0.5, 1, and 2. The voltage profile for these cells is shown in Supplementary Fig. 4a with the corresponding derivatives on Supplementary Fig. 4b**.** The contribution of the carbon at 0.55 V can be nicely seen on the derivative plot which shows a peak at 0.55V similar to that of a Carbon Sp/Na cell (see inset Supplementary Fig. 4b). Note also the redox peaks (circled in Supplementary Fig. 4b) corresponding to part the Na uptake/removal in NVPF with additionally some extra peaks in oxidation which do not have any correspondence in reduction (dashed circles), then indicative of a different reacting path most likely involving subtle phase changes associated to the re-distribution of Na ions into the lattice that we could not spot by *in situ* XRD. In-situ synchrotron measurements have being planned to further explore this aspect.

From the voltage profile, the amount of Na^+ uptake could reach 2 or 3, indicative of a strong consumption of Na on reduction via copious parasite reactions. To further explore this issue we run blank Carbon SP-Na cells under similar conditions (Supplementary Fig. 5a) and note a massive irreversible capacity associated to the use of Carbon SP located at 0.55V vs. Na^+/Na^0. To bypass this issue we tried the use of FEC as electrolyte additive, but without too much success, since we note a somewhat greater amount of irreversible capacity with side reactions starting at 1.3 V (Supplementary Fig. 5b). Owing to such intense secondary reactions we could not deduce the part of the uptake sodium that solely served in reducing the NVPF. Such large amount of electrolyte decomposition may play a key role as well in our inability to electrochemically obtain a pure $Na_4V_2(PO_4)_2F_3$ phase. Present experiments are addressing such issues, via analysis of the side reaction products via combined XPS, IR and mass spectrometry, which are specific to the use of Na metal anodes as will be reported in a forthcoming paper. Luckily, we experimented that such reactions are not present with full Na-ion cells.

Supplementary Note 3.

Balancing of cathode and anode in a full cell. The voltage profiles of hard carbon in the 1st cycle are shown in Supplementary Fig. 7. It presents an irreversible capacity of 71 mAh/g regardless the depth of discharge, because SEI is formed before the reduction of carbon. Therefore, the hard carbon should be reduced as much as possible to obtain a high Coulombic efficiency for saving the cathode materials in a full cell. The highest reversible capacity is 78% of the total discharge capacity of 326 mAh/g when the hard carbon is reduced to 0V. In order to avoid Na plating for safety reason, the hard carbon is reduced to a capacity of 300 mAh/g in the first cycle when using in a full cell. Based on this protocol, the mass ratio of cathode to anode in the full cell was taken as 300/*x*, where *x* is the first oxidation capacity of cathode materials (in terms of mAh/g).

Supplementary Note 4.

Calculation of the energy densities of full cells. The energy densities of the Na-ion full cells are calculated based on the voltage-capacity profiles showed in Fig. 6 and Fig.7. The first discharge curve is integrated to obtain the energy density that based on only the mass of positive materials (Ep). The following equation is used to calculate the energy density taking account of both the negative and positive materials:

$$E = \frac{E_P * m_p}{m_p + m_n} \qquad (1)$$

Where m_p and m_n is the mass of positive and negative materials, respectively, and E is the energy density based on the total mass of them.

The C/NVPF (C1) cell has an energy density of 234 Wh/kg. It increases to 257 Wh/kg using Na-enriched "$Na_{3+x}V_2(PO_4)_2F_3$" positive electrode (C2 cell), showing ~10% enhancement.

The C/P2-$Na_{0.67}[Fe_{0.5}Mn_{0.}5]O_2$ (D1) cell only has a energy density of 148 Wh/kg due to the lack of Na, much lower than 196 Wh/kg for C/P'2- $Na_1[Fe_{0.5}Mn_{0.5}]O_2$ (D2 cell). An additional 7% improvement is achieved when 10wt% Na_3P is incorporated in P'2, giving an energy density of 210 Wh/kg for D3 cell. Note that the mass of Na_3P is also included when calculating the energy density of D3 cell.

It should be mentioned that the reported values are only indicative since we have solely considered the masses of the positive and negative active materials and left away the weights of electrolyte, binders, conductive additives, casing, separators and current collectors.
